# Fortnightly atmospheric tides forced by spring and neap tides in coastal waters

**DOI:** 10.1038/srep10167

**Published:** 2015-05-18

**Authors:** Shinsuke Iwasaki, Atsuhiko Isobe, Yasuyuki Miyao

**Affiliations:** 1 Research Institute for Applied Mechanics, Kyushu University, 6-1 Kasuga-Koen, Kasuga 816-8580, Japan; 2 Department of Earth System Science and Technology, Kyushu University, 6-1 Kasuga-Koen, Kasuga 816-8580, Japan

## Abstract

The influence of sea surface temperature (SST) on atmospheric processes over the open ocean has been well documented. However, atmospheric responses to SST in coastal waters are poorly understood. Oceanic stratification (and consequently, SST) in coastal waters largely depends on the fortnightly spring–neap tidal cycle, because of variations in vertical tidal mixing. Here we investigate how changes in SST during the fortnightly tidal cycle affect the lower-level atmosphere over the Seto Inland Sea, Japan. We use a combination of *in situ* measurements, satellite observations and a regional atmospheric model. We find that the SST in summer shows cool (warm) anomalies over most of the inland sea during spring (neap) tides. Additionally, surface air temperature is positively correlated with the SST as it varies during the fortnightly tidal cycle. Moreover, the fortnightly spring–neap cycle also influences the surface wind speed because the atmospheric boundary layer becomes stabilized or destabilized in response to the difference between air temperature and SST.

Recent studies using satellite microwave remote sensing data have shown that surface winds are positively correlated with SST (i.e., stronger winds occur above warmer water) in open oceans, such as the tropics^1^,^2^, the Gulf Stream^3^,^4^,^5^, the Kuroshio^6^, the Kuroshio Extension^7^, and the northwestern Indian Ocean^8^, partly as a result of vertical momentum mixing with intense winds at high altitudes^9^. However, few studies have investigated the modification of surface winds by SST in coastal waters^10^,^11^. Studies of coastal waters may be limited by contamination of microwave remote sensing observations by surrounding land, coarse spatial resolution and a lack of *in situ* wind measurements above coastal waters. Nonetheless, especially in summer, it is likely that oceanic influences on atmospheric processes will be detected in coastal waters because intense tidal mixing causes large spatio-temporal variations in SST^12^.

The Seto Inland Sea was chosen for the present study. This semi-enclosed ocean surrounded by the Japan Islands includes narrow channels, islands and peninsulas (Supplementary Fig. 1) around which intense tidal mixing caused by large amplitude tidal currents is likely to destroy ocean stratification in summer. Additionally, the vertical mixing by tidal currents varies markedly with the fortnightly spring–neap cycle. It is likely that SST decreases (increases) as a result of strong (weak) vertical mixing because of the strong (weak) tidal currents during spring (neap) tides. Therefore, we expect that air temperature and/or surface winds also fluctuate with the fortnightly spring–neap tidal cycle (an atmospheric tide) in accordance with the tidally induced SST variations over the Seto Inland Sea during summer.

A composite analysis using Moderate Resolution Imaging Spectroradiometer (MODIS) infrared SST data from the Terra and Aqua satellites (see Methods) shows that SST varied with the spring–neap tidal cycle over the Seto Inland Sea in summer (June–August) during 2003–2012. The map of SST averaged over the entire period clearly shows cool waters in narrow straits where intense tidal mixing lowers SST (Fig. 1a). The composite differences between the averaged SST and the SST during spring (Fig. 1b) and neap (Fig. 1c) tides provide a more cogent explanation of the influence of tides. The SST during spring (neap) tides shows a cool (warm) anomaly over the entire Seto Inland Sea. This is as expected because strong (weak) vertical mixing is likely to occur during spring (neap) tides. This spring–neap cycle in ocean stratification as well as SST is a well-known coastal process associated with the intense tidal currents in the Seto Inland Sea. A previous study demonstrated that the intrusion of Kuroshio water into the upper water layer of the Seto Inland Sea occurs around neap tides as a result of the strong stratification (hence, weak vertical mixing)^13^.

Three numerical model experiments using the averaged SST, SST at spring tide and SST at neap tide demonstrated the tidal modulation of the lower-level atmosphere over the Seto Inland Sea. Regional atmospheric circulation modelling was conducted using these three SST datasets (see Methods for the model setup and Supplementary Fig. 1 for the model domain). One computation was forced by SST synthesised from multiple satellite observations irrespective of tidal cycles over the model domain (no tide run). The other two experiments were forced by the above- synthesised SST plus anomalies of MODIS SST at spring tides (Fig. 1b; spring tide run) and neap tides (Fig. 1c; neap tide run). Instead of forcing the atmosphere with an SST field that varies at a fortnightly frequency, the SST was kept constant in time for each experiment to obtain a clear mean, spring-tide or neap-tide response. Each response was representative of the actual atmospheric state at the corresponding fortnightly tidal phase (Supplementary Note 1 and Supplementary Fig. 2). This is because the local atmospheric boundary layer adjusts to the local SST within a few hours. Over most of the Seto Inland Sea the computed surface air temperature and wind speed were higher in the neap tide run than in the spring tide run (Fig. 2a,b; see Supplementary Fig. 3 for separate spring and neap tide runs). Surface wind speeds increase in a southwesterly direction along the Seto Inland Sea channel. The surface wind speed difference reaches about 7% (~0.25 s^−1^) in the western and central parts of the Seto Inland Sea.

The above intensification (weakening) of surface southwesterly winds during neap (spring) tides occurs as a result of vertical momentum mixing in the atmospheric boundary layer. In summer, at a height of 10 m, southerly and westerly winds prevail over the Seto Inland Sea (Fig. 3a). The wind direction at 1000 hPa height is also southerly and westerly (Supplementary Fig. 4a). These surface wind directions seem to be determined by the orographic conditions surrounding the Seto Inland Sea. Of particular interest is that, at heights of 950 and 925 hPa, southwesterly winds in the same direction as the surface intensification prevail (Supplementary Fig. 4b, c). Additionally, the wind speed increases with height because of sea-surface friction (Fig. 3a and Supplementary Fig. 4). Meanwhile, over most of the Seto Inland Sea the surface atmospheric stability, which is defined as air temperature at 1000 hPa height minus SST, becomes more unstable (<0) in the neap tide run than in the spring tide run (Fig. 3b and Supplementary Fig. 5 for separate tide runs). Therefore, it is suggested that the relatively high SST at neap tides lowers the surface atmospheric stability enhancing stronger vertical mixing. As a result, the momentum of the fast-moving upper-layer air is brought downward, and the southerly and westerly components of surface winds are accelerated^9^. The above process is confirmed by the fact that the surface wind difference between spring and neap tide runs is highly correlated (*r* = −0.96, significant at *P* < 0.01) with the difference in surface atmospheric stabilities between both models (Fig. 3c). The adjustment of sea level pressure may be considered as an alternative mechanism for the surface wind response to SST^14^. However, we did not find a significant correlation between the spring–neap differences in surface wind and the horizontal gradient of the sea level pressure (*r* = 0.17).

In agreement with the modelled results, *in situ* observational data also suggest fluctuations in the lower-level atmosphere with the fortnightly spring–neap cycle. First, we analysed SST, surface air temperature and wind speed data for summers from 1949 to 1996, archived by the Marine Information Research Center (MIRC) (see Methods). As observed by the MODIS satellite, SST in the Seto Inland Sea has a positive (negative) anomaly during neap (spring) tides (Fig. 4a and Supplementary Fig. 6a and b for each tide). Moreover, as shown in the regional atmospheric model, over most of the Seto Inland Sea surface air temperature (Fig. 4b) and wind speed (Fig. 4c) are higher during neap tides than during spring tides (see Supplementary Fig. 6c–f for each tide). Second, wind and SST observed at four moored buoys (Supplementary Fig. 1c) were also investigated. After removal of synoptic scale variations (Methods), time series averaged over the four stations demonstrated that the surface wind speed was mostly in phase with the SST variation with the fortnightly spring–neap cycle, with a positive and significant correlation coefficient of 0.56 on a *t*-test with 99% confidence levels (Fig. 5). A significant correlation between filtered surface wind and SST is also reported in western boundary current regions, such as the Kuroshio, North and South Atlantic and Agulhas Return Current^15^.

However, the MIRC data show that the differences in SST between neap and spring tides (Fig. 4a) are larger than the differences in MODIS data (Fig. 2c) by about 0.17 °C averaged over the Seto Inland Sea. This probably reflects the difference between the observation depths in these two datasets. The MIRC SST (conventionally referred to as a bulk SST) is measured on board using temperature sensors mounted beneath the uppermost surface layers. However, infrared radiometers mounted on satellites measure the skin temperature in the top ~10 μm of the attenuation depth of infrared radiation^16^. In summer, the difference in the skin temperature between neap and spring tides becomes moderate because of the intense surface heat flux. Additionally, the difference in air temperature between neap and spring tides in the MIRC data (Fig. 4b) is larger than the difference in the model simulations (Fig. 2a) by 0.4 °C on average. Although this underestimate in the modelled difference may have been caused by the choice of model schemes in the model setup (Methods), we did not investigate the technical requirements that might reduce the underestimation. Regardless, the larger difference in the observational data reinforces the idea that the lower-level atmosphere is modulated by the spring–neap tidal cycle.

Different from the modelled results, a significant correlation such as that shown in Fig. 3c could not be found between atmospheric stability and surface winds in the MIRC data. This is probably attributable to the inclusion of various time-scale variations unrelated to the fortnightly tidal cycle in the MIRC data, especially atmospheric properties. In the present study, Automated Meteorological Data Acquisition System (AMeDAS) surface wind speeds and air temperature were both band-pass filtered between 7 and 21 days to extract the signals with the fortnightly tidal cycle (Supplementary Note 2 and Supplementary Fig. 7). In addition, the National Centers for Environmental Prediction (NCEP)-reanalysis data wind speeds were removed from the buoy wind speeds to remove the synoptic-scale variation (Methods and Fig. 5). If these filters were not applied to AMeDAS and buoy data, no significant correlation (particularly wind speed) could be found between SST and atmospheric properties. Unfortunately, these filters cannot be applied to temporally interrupted MIRC data obtained sporadically in time. In addition, the following four points could explain the lack of a significant correlation. First, there are limited SST and atmospheric properties archived in MIRC data, and the available data may not be sufficient to identify a significant correlation. Second, MIRC air temperature data are likely to be contaminated by a positive bias because of solar radiation reflected on ship decks. Third, the MIRC dataset provides the bulk SST, but skin temperature is required to compute stability in the lower-level atmosphere. Fourth, estimation errors were possibly added to the MIRC wind speed data in the conversion from the Beaufort scale to wind speed.

It should be noted that the difference in surface winds with the fortnightly tidal cycle (7% in Fig. 2b) depends on the definition of the spring and neap tides. In the present study, the spring (neap) tides are defined as occurring over 3–4 days around the new/full (half) moon (see Method). Here, let us consider an extreme case for comparison. The change in SST related to the spring (neap) tides increases when the spring (neap) tides are defined as occurring only on the day of the new/full (half) moon (Supplementary Fig. 8). The modelled air temperature and surface winds vary considerably with these two SST datasets (Supplementary Note 3 and Supplementary Fig. 9). The surface wind speed becomes higher in the neap tide run than in the spring tide run by about 12% (~0.6 m s^−1^) in the western and central parts of the Seto Inland Sea (Supplementary Fig. 9c).

The present study identifies an atmospheric tide. It should be noted that the atmospheric tide described here is not the well-known planetary-scale tide that occurs as a direct consequence of atmospheric heating by solar radiation and/or tide generating forces. Instead, it is generated regionally by SST fluctuations with the fortnightly spring–neap cycle. This atmospheric tide is likely to occur, not only on the Seto Inland Sea, but also on various coastal waters around the world because intense tidal currents and tidal mixing are a universal feature in shallow coastal waters with complicated geography. Therefore, the lower-level atmosphere over coastal waters becomes relatively cool and calm (warm and windy) during spring (neap) tides.

The influences of the fortnightly atmospheric tide above coastal waters spread to surrounding coastal lands. In fact, in accordance with SST variations, surface air temperature has a negative (positive) anomaly during spring (neap) tides on the landmass around the Seto Inland Sea (Supplementary Note 2 and Supplementary Fig. 7a, b). In addition, surface wind speeds also decrease (increase) in a southwesterly direction during spring (neap) tides along the coast of the Seto Inland Sea (Supplementary Fig. 7c, d). In the present study, all anomalous fields can be regarded as composites of various events. In fact, this SST-induced coastal atmospheric tide does not always appear in the Seto Inland Sea. The atmospheric processes over the coastal waters are likely to be dependent on large-scale atmospheric phenomena as well as tidally fluctuating SST.

Finally, the fact that SST is influenced by the atmosphere via surface heat flux is well known, whereas we have demonstrated that SST variability in coastal waters influences the atmosphere. This leads to the possibility of two-way coupling between the atmosphere and coastal waters. In addition to the local (vertical) processes we have explored in this study, SST anomalies can be generated by anomalous horizontal advection caused by oceanic surface-velocity anomalies, which in turn are driven by wind anomalies. In our model experiments, the mean surface heat flux in the Seto Inland Sea was higher by ~60 W m^−2^ at neap tides than at spring tides (Supplementary Fig. 10) because of a combination of SST, air-temperature and surface-wind changes. This heat flux anomaly is as large as 72% of the mean summer heat flux. If feedbacks onto the sea were included, this anomalous heat flux would certainly change SST, especially because the Seto Inland Sea is shallow (average depth 30 m). Therefore, regional atmosphere–ocean coupling models^11^,^17^ may improve our understanding of atmosphere–ocean processes in the inland sea.

## Methods

MODIS SST data for the Seto Inland Sea from June to August (summer) for 2003–2012 were provided by the Japan Aerospace Exploration Agency and Tokai University from the website http://www.eorc.jaxa.jp/hatoyama/satellite/sendata/modis_j.html. The SST data were sorted by spring and neap tides to produce the SST maps averaged during each tide. Following the method of the Japan Coast Guard, spring and neap tides were defined as lunar ages of 29 and 0–2 (also, 14–17), and of 7–9 (also, 22–24), respectively. The daily data for lunar age were downloaded from the website of the Chronological Scientific Tables (https://www.rikanenpyo.jp/index.html). Lunar ages are defined at Japan Standard Time (JST). In the present study, decimals in lunar ages were omitted. The times (UTC) recorded in MODIS SST and MIRC datasets were changed to JST when these datasets were sorted by spring and neap tides.

We used the Pennsylvania State University–National Center for Atmospheric Research (NCAR) Mesoscale Model (MM5V3)^18^ in a domain from 115°E to 150°E, 22°N to 45°N (Supplementary Fig. 1a) with a horizontal resolution of 10 km and 23 sigma levels in the vertical. The NCEP Final Operational Global Data (http://rda.ucar.edu/datasets/ds083.2/) were used for the initial and lateral boundary conditions. Land-surface temperature was predicted using a five-layer soil model based on the vertical diffusion equation (ISOIL = 1 in the MM5 user’s guide^19^). The Grell cumulus parameterization (ICUPA = 3), medium-range forecast planetary boundary layer (IBLTYP = 5), cloud and rainwater (IMPHYS = 4) and cloud radiation (IFRAD = 2) schemes were also included in the model. The radiative condition (IFUPR = 1) was chosen for the upper boundary (100 hPa level) to absorb upward-propagating wave momentum. The modelled levels at 1000, 950, 925, 900 and 850 hPa were regarded as the atmospheric boundary layer because, in general, the boundary layer develops at a height of 200–1500 m height over the Seto Inland Sea^20^. For the no tide run, Multiscale Ultrahigh Resolution Sea Surface Temperature (MURSST) data^21^ downloaded from the Jet Propulsion Laboratory website (http://mur.jpl.nasa.gov/index.php) were averaged over the period from 1 June to 31 August from 2003 to 2012, and were averaged within each grid cell of the MM5V3 to impose the boundary condition at the sea surface. The above-averaged MURSST plus MODIS SST anomalies at spring and neap tides were added over the Seto Inland Sea for the spring (Supplementary Fig. 3a) and neap tide runs (Supplementary Fig. 3b), respectively. Although an invariant SST distribution was given to the model, in reality SST varies in time with the fortnightly spring–neap cycle. This simplification is justified, as mentioned in the text, by the quick response of the atmospheric boundary layer in the actual situation (Supplementary Note 1). The MODIS data were used for the spring and neap tide runs because the MURSST data are overly smoothed in time, and because the amplitude of the fortnightly cycle is considerably reduced. Computations from 16 May to the end of August were conducted separately for ten summer seasons from 2003 to 2012. Model results were saved every 6 hours from 1 June to the end of August.

The MIRC (http://www.mirc.jha.or.jp/) archives air temperature and wind speed data measured on research vessels in addition to SST obtained by various organizations conducting operational ocean observations. We used these data for the Seto Inland Sea for June to August from 1949 to 1996 after conducting quality control as follows. First, the present study did not use these hydrographic data, but used wind force on the Beaufort scale (more than 80%) or wind speeds (less than 20%), plus wind directions measured with anemometers on board research vessels. The Beaufort scale data were only used when they were provided with wind directions because data collected by visual wind observations are considerably less reliable. The wind force was replaced with the median wind speed within each Beaufort scale range. Second, we removed data that exceeded three times the standard deviation from the climatological monthly mean of the data within each 0.2° grid cell. Third, we converted wind speeds to speeds at a height of 10 m according to the logarithmic law^22^. Because the MIRC dataset does not include the anemometer height of each individual ship, we used 8.3 m as the anemometer height, which is an average for research vessels currently operating in the Seto Inland Sea. As for the MODIS data, the wind speed data were sorted by spring and neap tides to produce the averaged maps.

The buoy data over the eastern part of the Seto Inland Sea (Supplementary Fig. 1c) were downloaded from the Ministry of Land, Infrastructure, Transport and Tourism website (http://222.158.204.199/obweb/). We used SST at a depth of 5 m (as the bulk SST), wind speed and wind directions observed at four buoys. First, wind speeds observed at all buoys were adjusted to those at a 10-m height (defined as surface wind) in the same manner as the MIRC data. Second, the daily zonal/meridional wind speeds and SST data were averaged for each buoy. The SST and wind data for the four buoys were then averaged to use in the subsequent analyses. Third, because southwesterly winds prevail at the buoy stations during summer, surface wind speeds in the southwest-northeast direction were extracted for the analyses. Fourth, the daily mean wind speeds reproduced in the NCEP-NCAR reanalysis data^23^ at the nearest grid point were removed from the buoy-derived wind speeds. This was done because synoptic-scale variations induced by SST independent of locality are anticipated to diminish by removing the relatively coarse reanalysis data (with a resolution of 2° in both latitude and longitude). Fifth, surface wind speeds and SST were both band-pass filtered between 7 and 21 days using a boxcar filter to extract the signals with the fortnightly spring–neap tidal cycle.

## Author Contributions

S.I. and A.I. designed and performed the model experiments. S.I. supervised the work. Y.M. carried out the processing of the MIRC Ocean dataset. S.I. analysed the observational data and model simulations. S.I. and A.I. wrote the manuscript.

## Additional Information

**How to cite this article**: Iwasaki, S. *et al.* Fortnightly atmospheric tides forced by spring and neap tides in coastal waters. *Sci. Rep.* **5**, 10167; doi: 10.1038/srep10167 (2015).

## Supplementary Material

Supporting Information

## Figures and Tables

**Figure 1:**
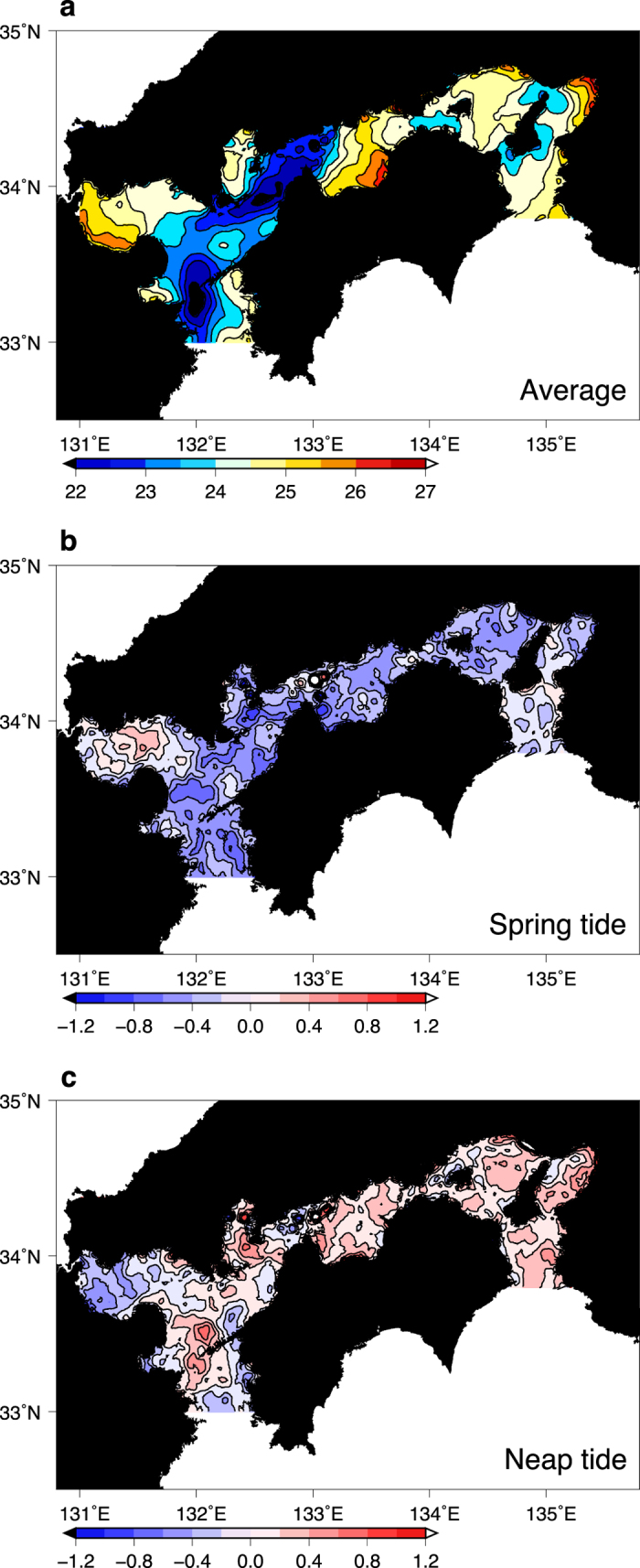
Satellite-observed SST variation with the fortnightly spring–neap tidal cycle over the Seto Inland Sea. **a–c**, (**a**) MODIS SST averaged over summers (June–August) from 2003 to 2012 and anomalies from the average during the (**b**) spring or (**c**) neap tides. The data were smoothed with a two-dimensional boxcar filter with a width of 6 km in each direction. Values are represented by colour shading as shown in the scale below each panel. Contour intervals are (**a**) 0.5 °C, (**b**) 0.2 °C, and (**c**) 0.2 °C. The Generic Mapping Tools were used to create the maps in this figure.

**Figure 2:**
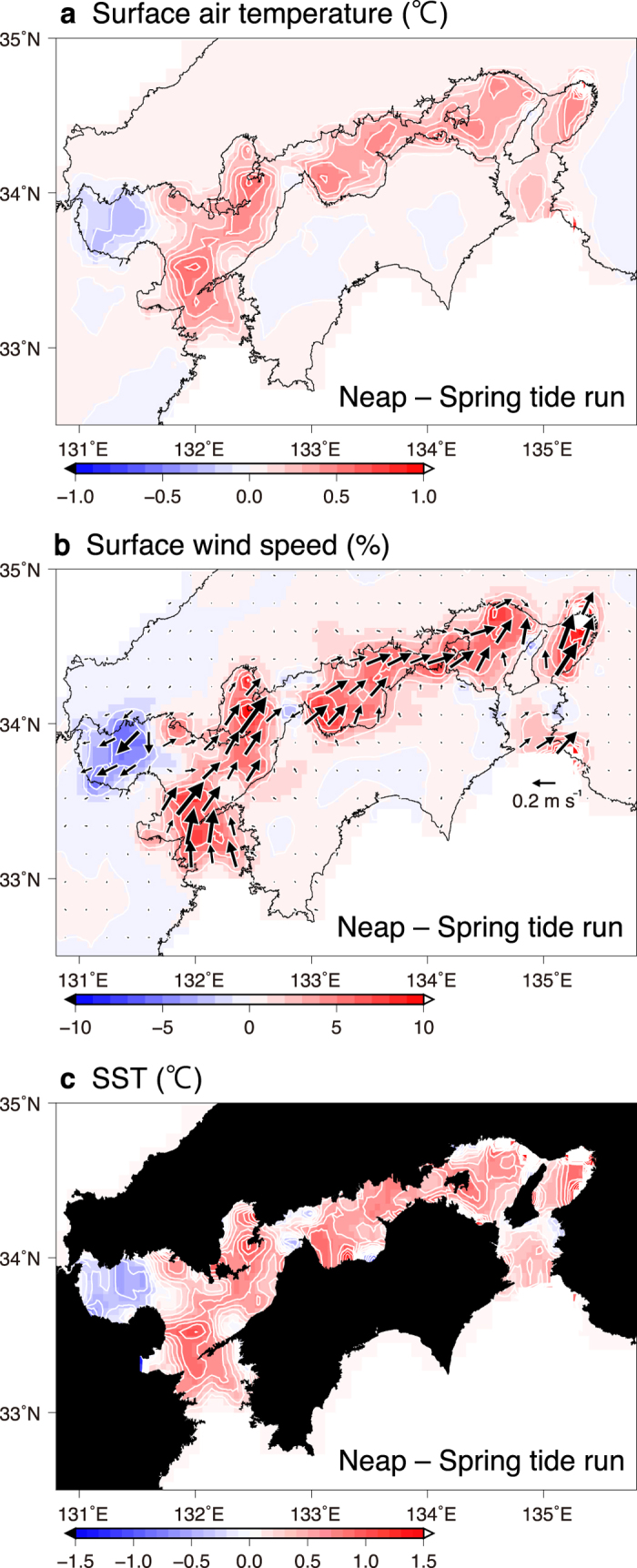
Simulated differences in surface atmospheric properties. Values of the neap tide run minus the spring tide run are shown by colour shading with contours. **a–c**, (**a**) modelled air temperature (2-m height), (**b**) modelled wind speed (10-m height), and (**c**) MODIS SST imposed on the modelled sea surface. All values and wind vectors are averaged over summers (June–August) from 2003 to 2012. The scales are shown at the bottom of each panel. Contour intervals are (**a**) 0.1 °C, (**b**) 2%, and (**c**) 0.15 °C. In panel (**b**) the colour shading represents the ratio of the wind-speed difference between spring and neap tide runs to wind speeds in spring tide runs. The Generic Mapping Tools were used to create the maps in this figure.

**Figure 3:**
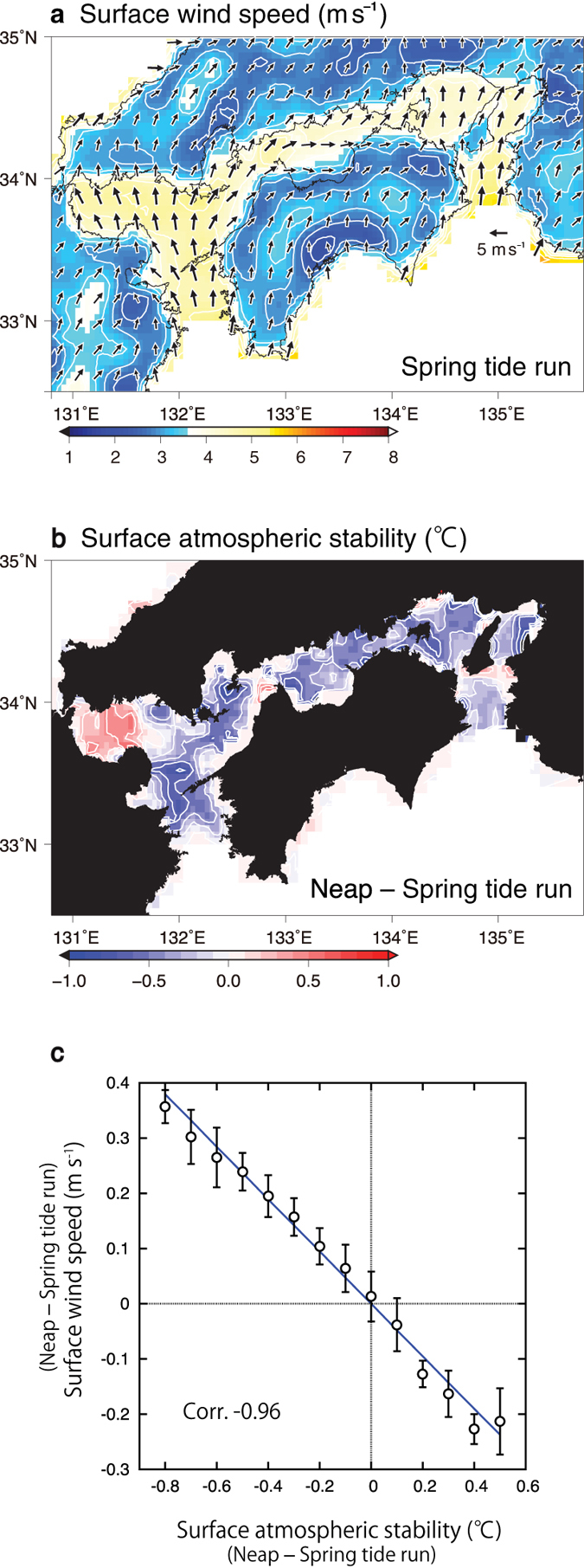
Modelled surface wind and surface atmospheric stability during summer. **a**–**c**, All values are averaged over summers (June–August) from 2003 to 2012.(**a**) Wind fields at 10 m in the spring tide run, and (**b**) surface atmospheric stability difference between spring and neap tide runs. Vectors indicate winds with scales shown in the middle right of the panel. See the text for the definition of the surface atmospheric stability in (**c**). Contour intervals are (**a**) 0.4 m s^−1^ and (**b**) 0.2 °C. **c**, Scatter diagram between surface atmospheric stability versus wind speeds at 10-m height. The data are differences between neap and spring tide runs, and are averaged over summers (June–August) from 2003 to 2012 at each grid cell over the Seto Inland Sea. Open circles indicate mean wind-speed difference values within each bin of the stability difference with the interval of 0.1 °C, and the error bars represent ± one standard deviation in each bin. The blue line indicates the regression line. The correlation coefficient is shown in the lower left corner of the panel (significant at *P* < 0.01). The correlation coefficient (–0.96) was computed using data before the binned average (n = 689). The Generic Mapping Tools were used to create the maps in (**a**) and (**b**). Gnuplot was used to create the graph in (**c**).

**Figure 4:**
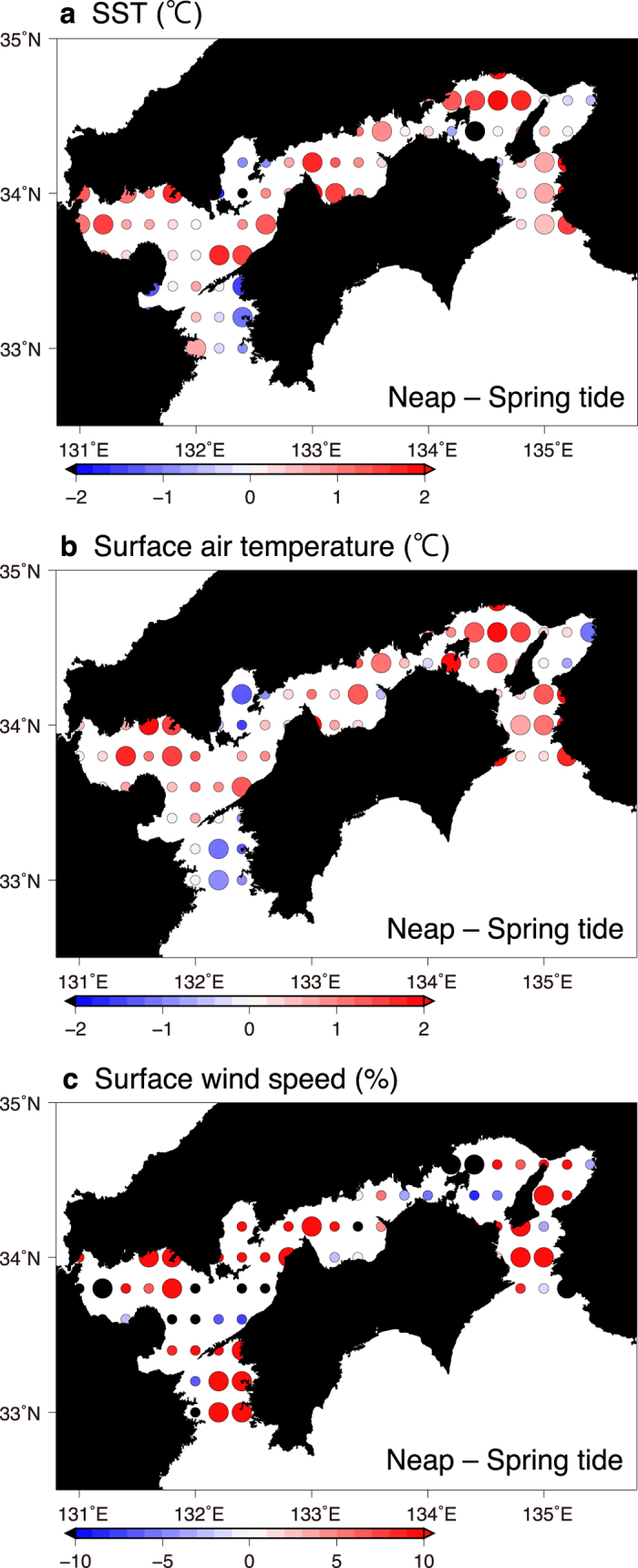
Same as for Fig. 2, but for observed summer properties archived in the MIRC dataset from 1949 to 1996. See Methods for data processing of the MIRC dataset. The difference between neap and spring tides is shown by circles with colour shading using the scale shown at the bottom of each panel. The large (small) circles are used for values significant (insignificant) on a *t*-test with 95% confidence levels. In panel (**c**), the colour shading represents the ratio of the wind-speed difference between spring and neap tides to wind speeds at spring tides. The Generic Mapping Tools were used to create the maps in this figure.

**Figure 5:**
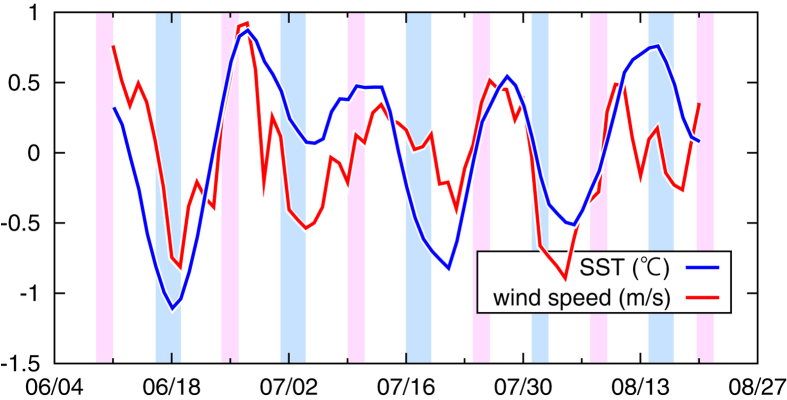
Time series of the wind speed (red curve) and SST (blue curve) observed at buoy stations during summer 2011. The abscissa indicates month/day. The blue and red shaded periods denote the spring and neap tide phases, respectively. Both time series are band-pass filtered using a boxcar filter between 7 and 21 days. Gnuplot was used to create the graph in this figure.
