# Supplementary material for: Fortnightly atmospheric tides forced by spring and neap tides in coastal waters
Source: Sci Rep. 2015 May 18;5:10167. doi: 10.1038/srep10167 (PMC4435005; doi:10.1038/srep10167)
Supplement: Supporting Information [file srep10167-s1.pdf]

SUPPLEMENTARY INFORMATION

Fortnightly atmospheric tides forced by spring and neap tides in coastal waters

Shinsuke Iwasaki[1], Atsuhiko Isobe[1] and Yasuyuki Miyao[2]

[1]*Research Institute for Applied Mechanics, Kyushu University, 6-1 Kasuga-Koen, Kasuga 816-8580, Japan*
[2]*Department of Earth System Science and Technology, Kyushu University, 6-1 Kasuga-Koen, Kasuga 816-8580, Japan*

Supplementary Notes

1. Timescale of atmospheric responses to SST fluctuated with a fortnightly tidal cycle.

We conducted an extra model experiment to investigate the timescale of atmospheric responses to SST fluctuated continuously with a fortnightly tidal cycle over the Seto Inland Sea. Although the numerical experiment with SST fixed in time has an advantage in that computations are conducted under many different atmospheric conditions (Methods), this experiment is not justified unless the experiment with SST varying in time provides similar results. The model experiment was forced by the synthesised SST[S1] plus the SST fluctuations with a fortnightly tidal cycle (Supplementary Fig. 2a; tide run). The amplitude of the SST fluctuations with the fortnightly tidal cycle was equal to the differences in MODIS SST at spring and neap tides shown in Supplementary Fig 8a and b, respectively. Computations from 16 May to the end of August in 2012 were conducted in the present experiment. In addition to the tide run, an additional experiment was conducted using SST after removal of tidal fluctuations (no tide run; see blue dashed line in Supplementary Fig. 2a) to compute synoptic-scale variations unrelated to SST varying by tides. All conditions of MM5V3[S2], except SST over the Seto Inland Sea, and computation period are the same as those used in the three model experiments discussed in the text. Based on this model experiment, lag correlation coefficients between SST and meteorological properties (surface air temperature and wind speed after removal of those computed in the no tide run) have a

peak at hour 0 (Supplementary Fig. 2b). Therefore, atmospheric responses in the local atmospheric boundary layer are completed within a few hours, at least during summer in the Seto Inland Sea.

2. Analysis in the coastal region

To investigate whether the fortnightly atmospheric tide spreads to surrounding coastal lands, we analysed hourly data on surface wind speed and air temperature at Automated Meteorological Data Acquisition System (AMeDAS) stations (http://www.data.jma.go.jp/gmd/risk/obsdl/index.php) operated by the Japanese Meteorological Agency. The observation heights at AMeDAS stations are about 10 m for wind and 1.5 m for air temperature. In this study, we used the AMeDAS data from 110 sites surrounding the Seto Inland Sea from June to August during 2001 to 2009 (see Supplementary Fig. 7c and d for site locations). These AMeDAS sites were chosen subjectively so that they are distributed approximately evenly around the inland sea. The spring (neap) tides were defined as days just at the new/full (half) moon. The averages during the spring (neap) tides were computed using hourly data during each tide. Both the AMeDAS air temperature and wind data were band-pass filtered using a boxcar filter between 7 and 21 days for analysis with the fortnightly tidal cycle.

3. Two additional model experiments

We conducted two model experiments in addition to those described in the text and the experiment described in Supplementary Note 1. As described in the text, tide-induced SST anomalies increase when spring (neap) tides are defined as a single day just at the new/full (half) moon (Supplementary Fig. 8). These additional experiments were conducted to examine the extreme case for tidal modulation of the lower-level atmosphere, and were forced by the synthesised SST[S1] plus anomalies of MODIS SST at spring and neap tides as shown in Supplementary Fig. 8a and b, respectively. All conditions, except the Seto Inland Sea SST in the MM5V3[S2], were the same as those used in the three model experiments discussed in the text.

References

1. S1. Chin, T. M., Vazquez, J., Armstrong, E. M. & Mariano, A. J. Algorithm theoretical basis document: Multi-scale, motion-compensated analysis of sea surface temperature, version 1.1. NASA Measures Algorithm Theoretical Basis Doc., 17 pp. (2010). Available at: ftp://mariana.jpl.nasa.gov/mur_sst/tmchin/docs/ATBD/atbd_1.1actual.pdf. (Accessed: 16th January 2015)

2. S2. Grell, G. A., Dudhia, J. & Stauffer, D. A description of the fifth-generation Penn State/NCAR mesoscale model (MM5). NCAR Tech. Note NCAR/TN-398+STR, 117 pp. (1994). Available at: http://nldr.library.ucar.edu/repository/assets/technotes/TECH-NOTE-000-000-000-214.pdf. (Accessed: 16th January 2015)

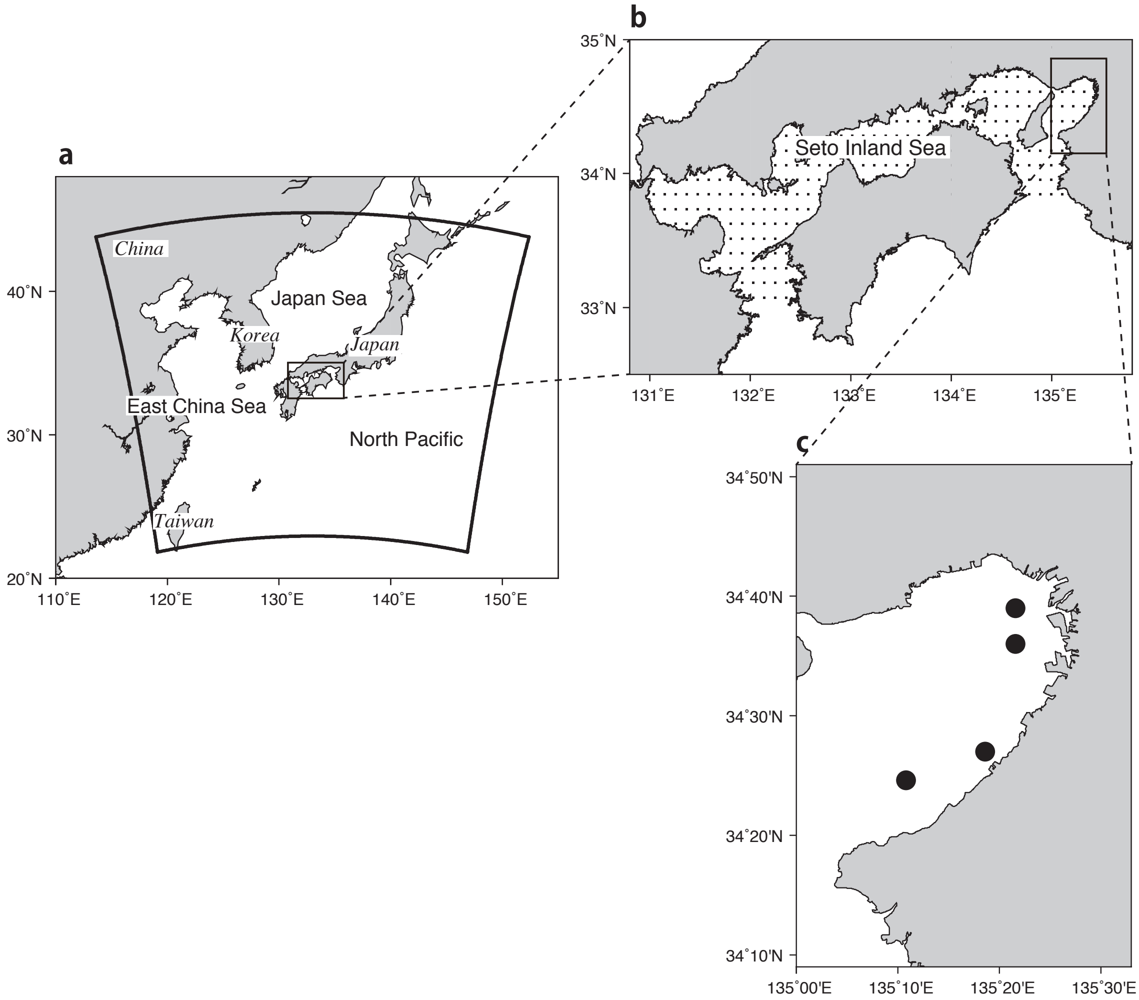

Supplementary Figure 1: Model domain. a, The bold line shows the domain of the regional atmospheric model adopted in the present study. **b**, The area within the small box in panel (**a**) is enlarged to show the Seto Inland Sea. MODIS SST anomalies are given to the central point of each grid cell (dots) for surface boundary conditions of neap and spring tide runs. **c**, The area within the small box in panel (**b**) is enlarged to show the positions of the buoys (closed dots). The Generic Mapping Tools were used to create the maps in this figure.

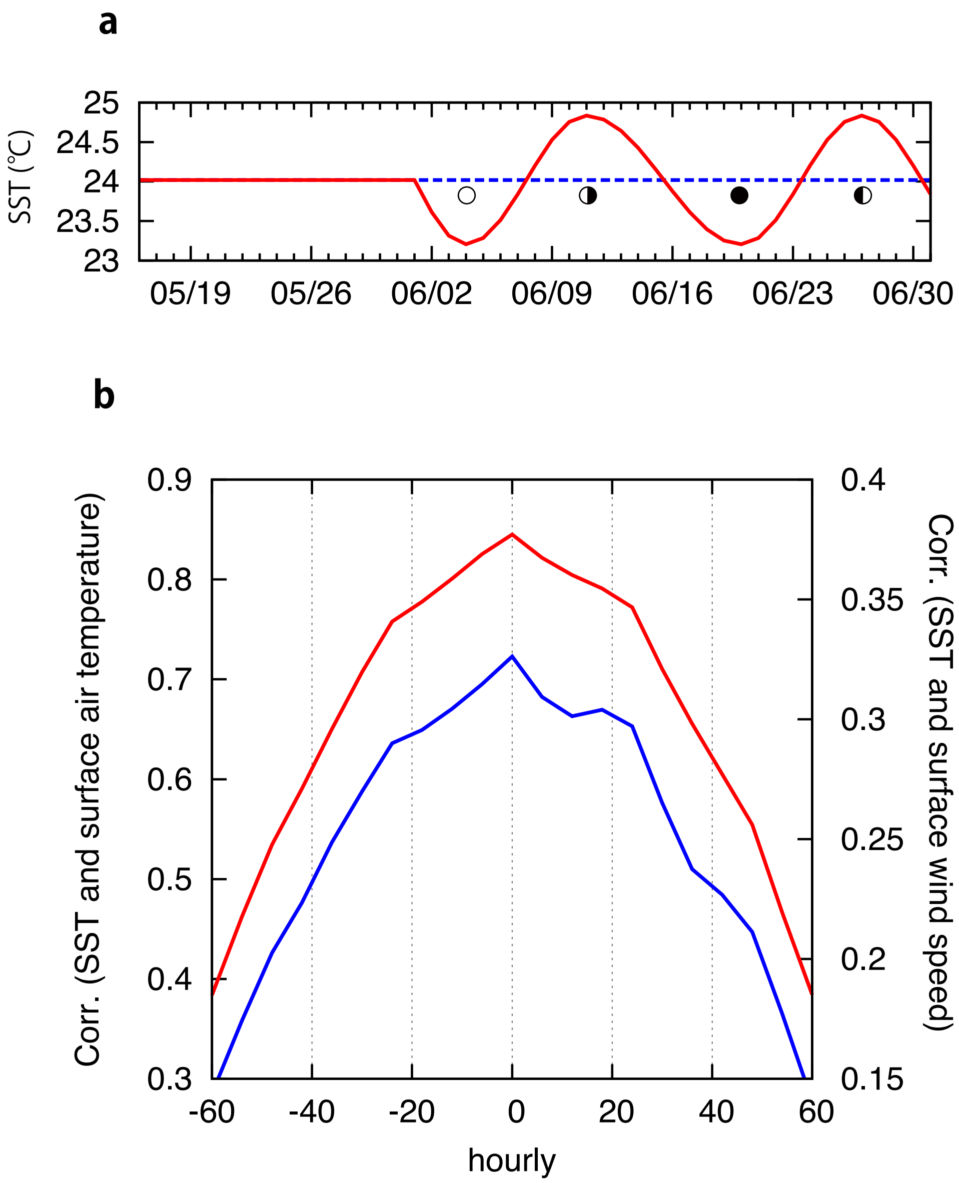

Supplementary Figure 2: Timescale of atmospheric responses to SST fluctuated with a fortnightly tidal cycle. a, An example of time series of SST for the tide (red curve) and no tide (blue broken line) runs at 33.3°N and 132°E. The abscissa indicates month/day. The open, closed (i.e., spring tides) and half open (i.e., neap tides) circles indicate full, new and half moons, respectively. **b,** Lag correlations between SST and surface air temperature (red curve) and between SST and surface wind speed (blue curve). The differences between tide and no tide runs were used as the atmospheric properties to remove synoptic scale variations, and were averaged at each grid cell over the Seto Inland Sea. Positive lags mean that SST varies in time prior to surface air temperature and wind speed. Gnuplot was used to create the maps in this figure.

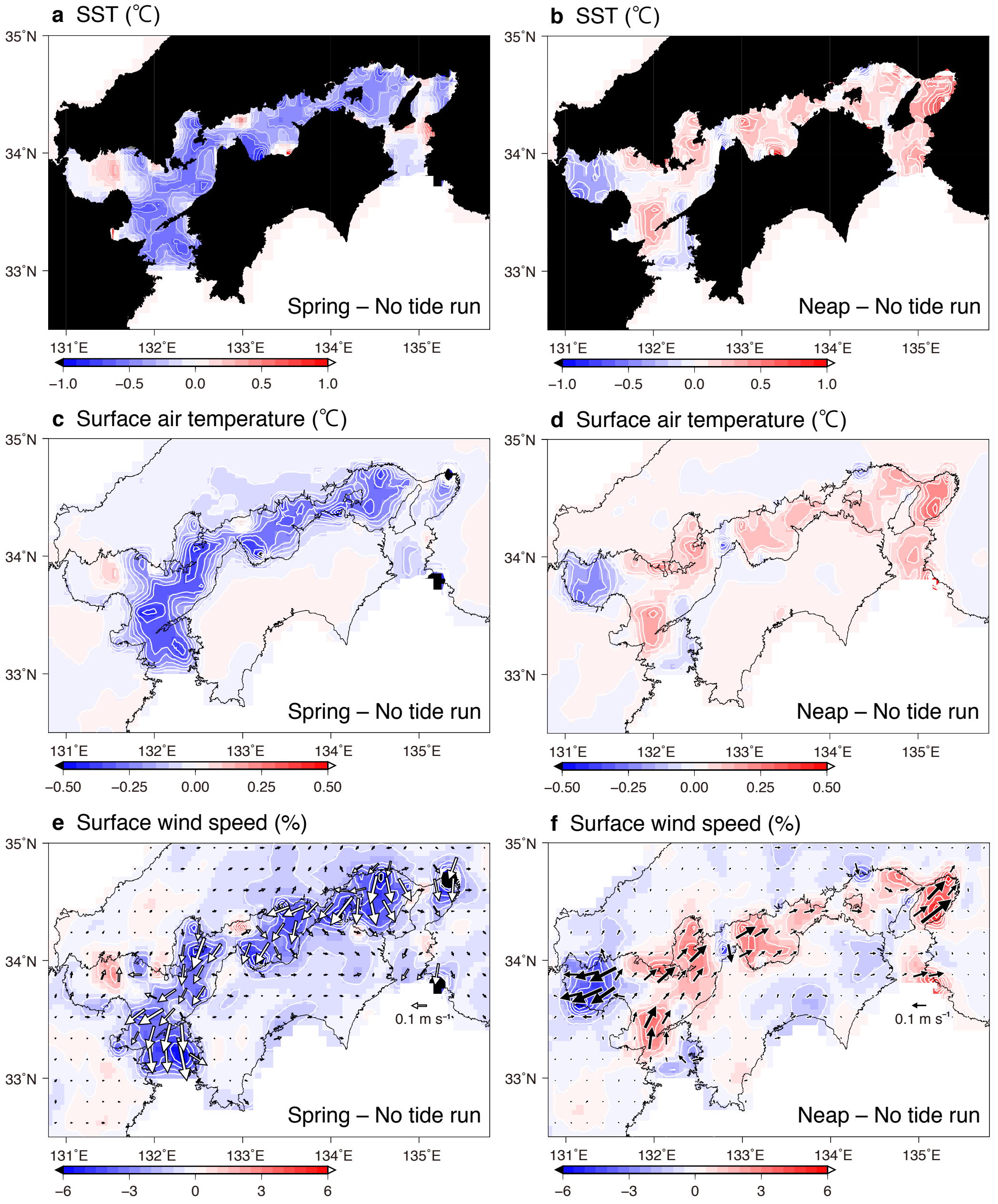

Supplementary Figure 3: Simulated differences in surface atmospheric properties. Values shown are the spring tide run minus the no tide run (left panels), and the neap tide run minus the no tide run (right). a–f, (**a, b**) MODIS SST, (**c, d**) air temperature at 2-m height and (**e, f**) wind speeds at 10-m height, which were all averaged over summer (June–August) from 2003 to 2012. The scales are shown at the bottom of each panel. Contour intervals are (**a, b**) 0.1 °C, (**c, d**) 0.05 °C and (**e, f**) 1%. In panels (**e**) and (**f**), the colour shading represents the ratio of the wind-speed difference from the no tide run to the wind speed in the no tide run. The Generic Mapping Tools were used to create the maps in this figure.

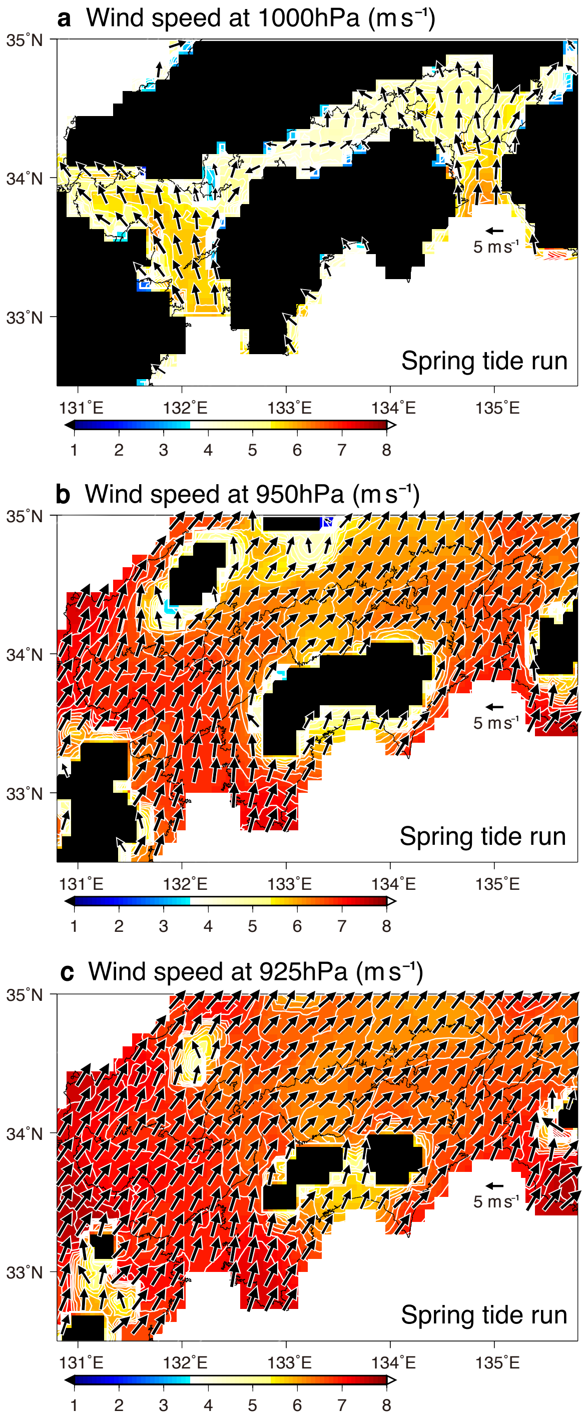

Supplementary Figure 4: Modelled wind field in the spring tide run during summer. a–c, All values are averaged over summers (June–August) from 2003 to 2012. Wind fields are shown at (**a**) 1000 hPa, (**b**) 950 hPa and (**c**) 925 hPa in the spring tide run. Vectors indicate winds with scales shown in the middle right of each panel. Contour intervals are 0.2 m s^{-1} for all panels. The Generic Mapping Tools were used to create the maps in this figure.

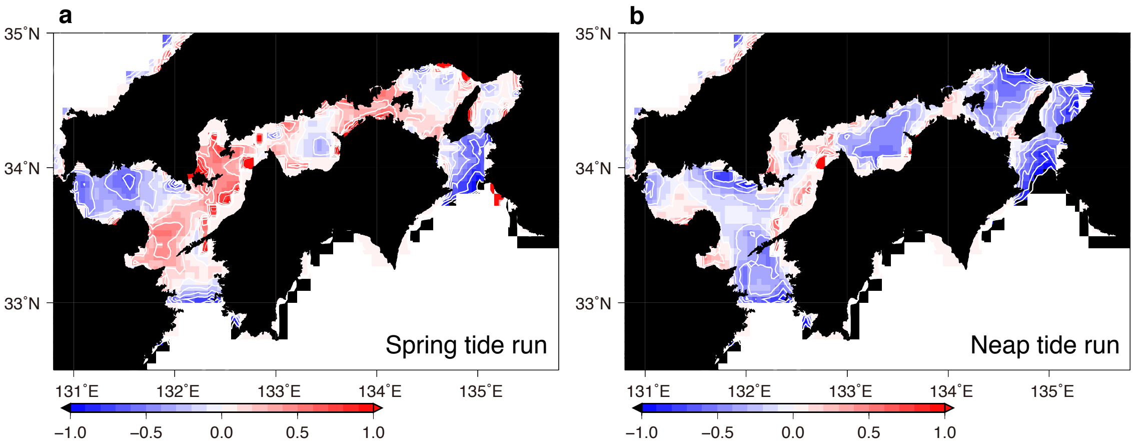

Supplementary Figure 5: Modelled surface atmospheric stability during summer. **a, b,** All values are averaged over summers (June–August) from 2003 to 2012. (**a**) Surface atmospheric stability in the spring tide run and (**b**) stability in the neap tide run. See the text for the definition of the surface atmospheric stability. Contour intervals are (**a, b**) 0.2 °C. The Generic Mapping Tools were used to create the maps in this figure.

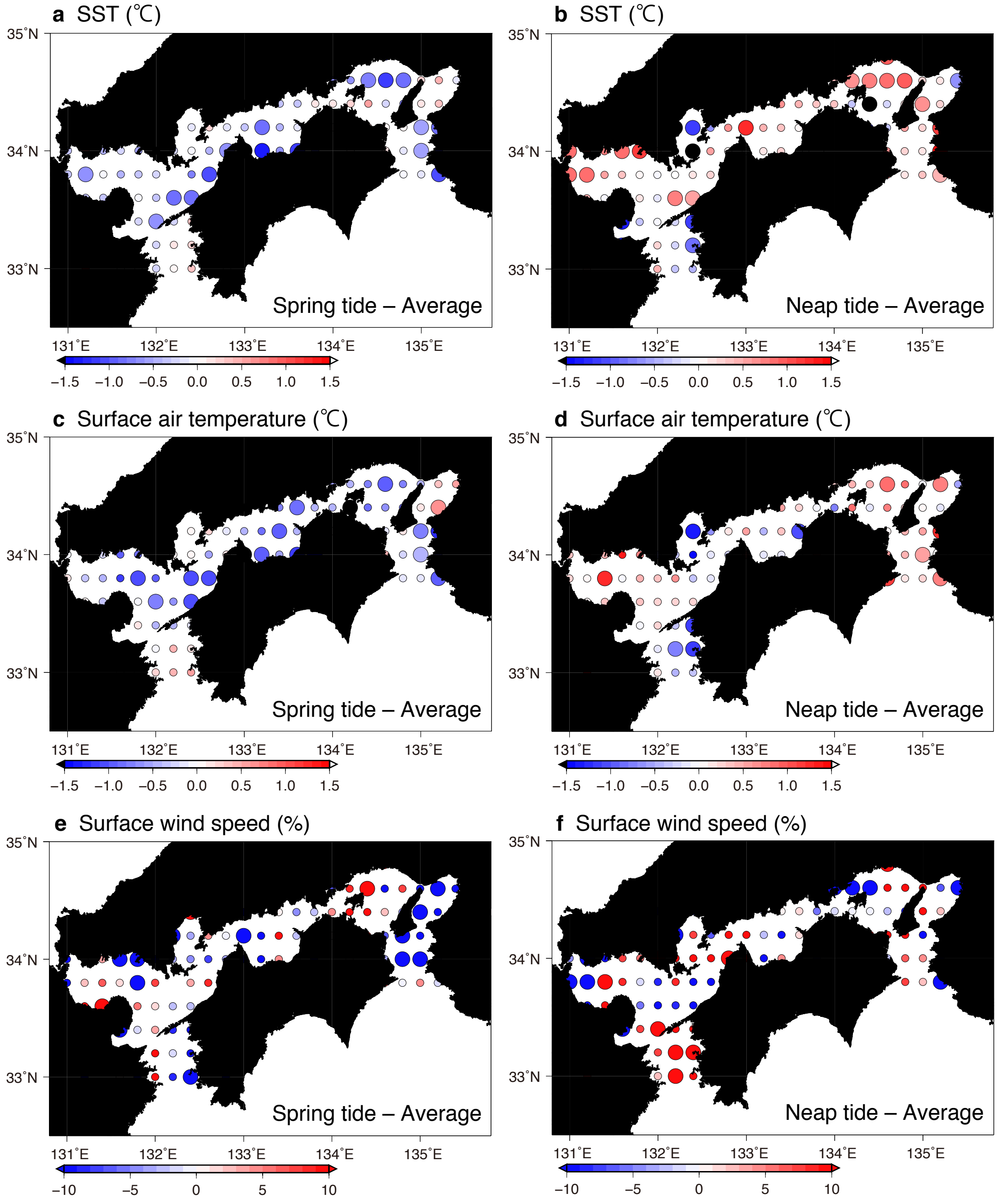

Supplementary Figure 6: Same as for Supplementary Fig. 2, but for observed summer properties archived in the MIRC dataset from 1949 to 1996. See Methods for data processing of the MIRC dataset. The scales are shown at the bottom of each panel. The large (small) circles are used for values significant (insignificant) on a *t*-test with 95% confidence levels. In panels (**e**) and (**f**), the colour shading represents the ratio of anomalous wind speed from wind speed averaged over summers to the wind speed averaged over summers. The Generic Mapping Tools were used to create the maps in this figure.

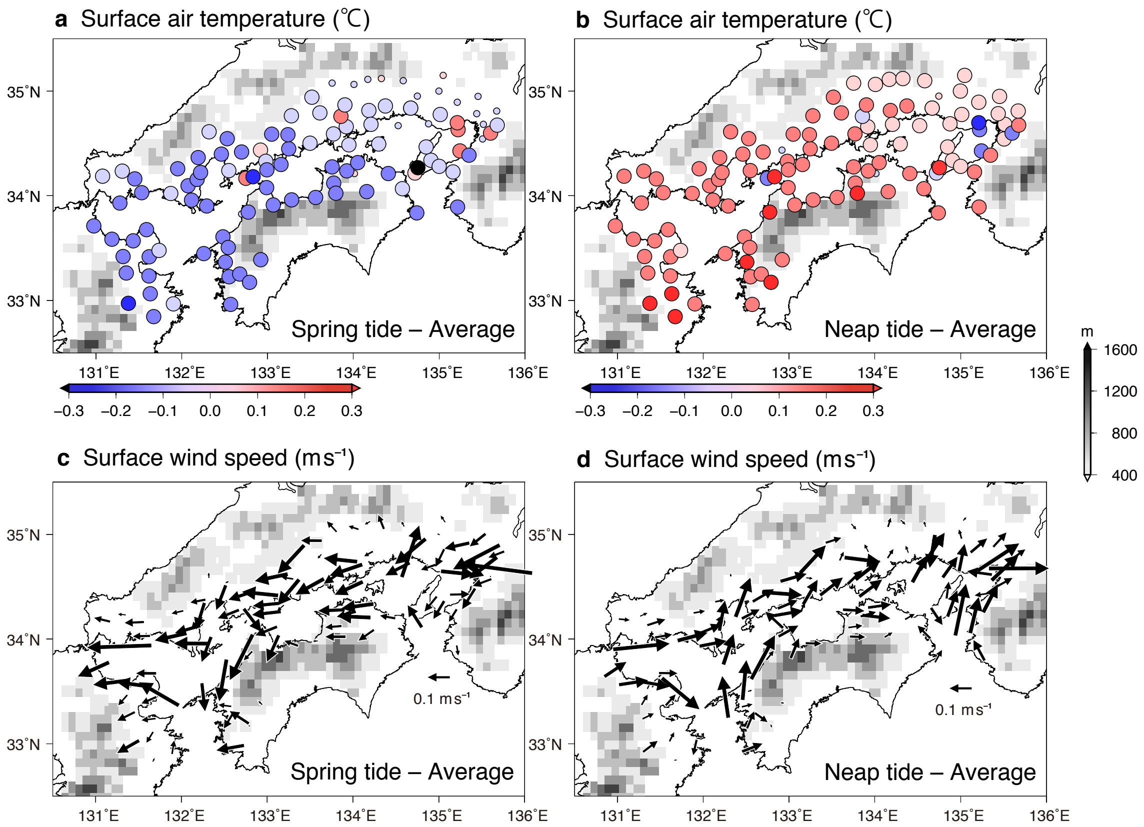

Supplementary Figure 7: Observed surface air temperature and wind anomalies with the fortnightly spring–neap tidal cycle over the coastal lands adjacent to the Seto Inland Sea. AMeDAS surface air temperature (upper two panels) and wind vectors (lower two panels) are averaged only at spring (panels **a** and **c**) and neap (**b** and **d**) tides, respectively. Anomalous air temperature and wind from those averaged over summers are depicted in (**a**–**d**). The scales of air temperature are shown at the bottom of panels **a** and **b**. The large (small) circles are used for values significant (insignificant) on a *t*-test with 95% confidence level. The vectors are represented for values significant on a *t*-test with 95% confidence level. Grey shading on the land in (**a**–**d**) shows topographic height in metres. The Generic Mapping Tools were used to create the maps in this figure.

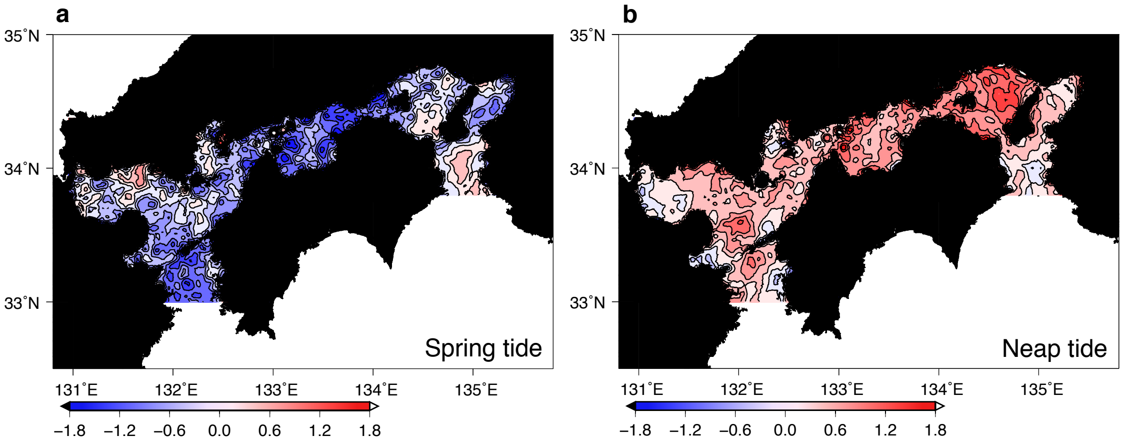

Supplementary Figure 8: Same as for Fig. 1, but for the spring (neap) tides defined as one day at the new/full (half) moon. Summer SST anomalies at (**a**) spring and (**b**) neap tides. (The mean summer SST is shown in Fig. 1**a**). The data were smoothed with a two-dimensional boxcar filter with a width of 6 km in each direction. Values are represented by colour shading as shown in the scale below each panel. Contour intervals in panels **a** and **b** are both 0.3 °C. The Generic Mapping Tools were used to create the maps in this figure.

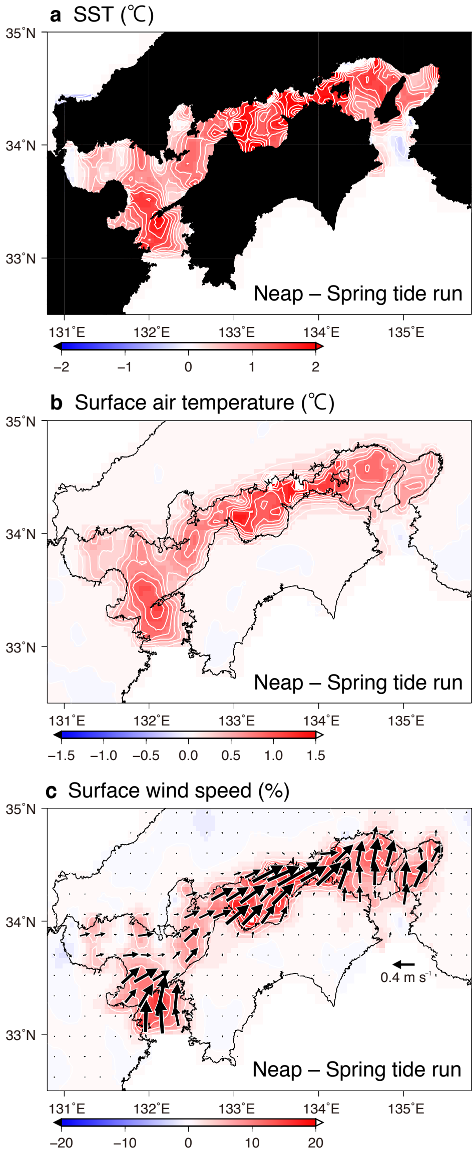

Supplementary Figure 9: Same as for Fig. 2, but for sea surface boundary conditions. The two experiments were forced by the synthesised SST plus anomalies of MODIS SST at spring tides (Supplementary Fig. 8**a**; spring tide run) and neap tides (Supplementary Fig. 8**b**; neap tide run). **a**–**c**, (**a**) MODIS SST imposed on the modelled sea surface, (**b**) modelled air temperature (2-m height), and (**c**) modelled wind speed (10-m height). All values and wind vectors are averaged over summers (June–August) from 2003 to 2012. The scales are shown at the bottom of each panel. In panel (**c**), the colour shading represents the ratio of the wind-speed difference between spring and neap tide runs to wind speeds in spring tide runs. Contour intervals are (**a**) 0.2 °C, (**b**) 0.15 °C, and (**c**) 4%. The Generic Mapping Tools were used to create the maps in this figure.

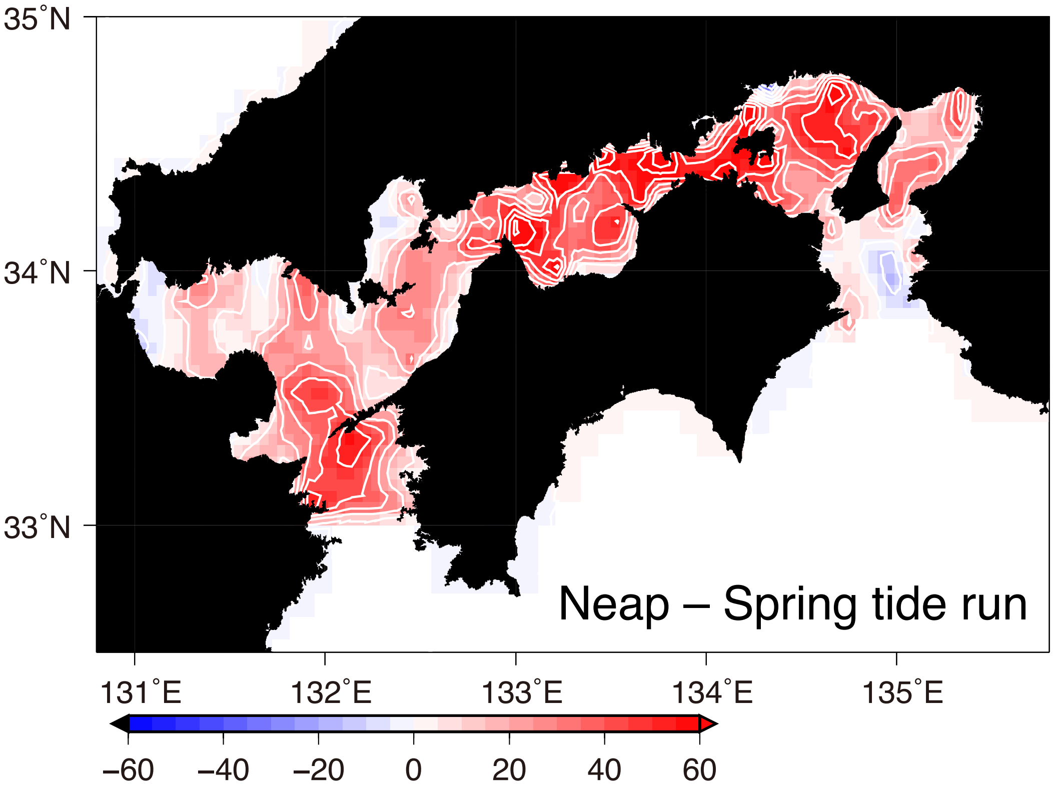

Supplementary Figure 10: Difference in the modelled surface heat flux between neap and spring tides (neap tide run minus spring tide run). The two experiments were forced by the synthesised SST plus anomalies of MODIS SST at spring tides (Supplementary Fig. 8**a**; spring tide run) and neap tides (Supplementary Fig. 8**b**; neap tide run). The surface heat flux is defined as the sum of latent heat and sensible heat fluxes, which were averaged over summers from 2003 to 2012. Positive values indicate that the upward (ocean to atmosphere) heat flux in the neap tide run was larger than that in the spring tide run. Contour interval is 10 W m^{-2}. The Generic Mapping Tools were used to create the map in this figure.
